# Ammoniuni-Bromatum in Bronchial Troubles

**Published:** 1896-05

**Authors:** H. Goullon


### AMMONIUM-BROMATUM IN BRONCHIAL TROUBLES.

DR. H. GOULLON.

(Translated by the late Dr. Samuel Lilienthal.)

An aged lady, past sixty, suffered since January from extensive bronchial catarrh; rattling murmurs all over chest, with most difficult expectoration; cough very painful, so that she has to support the chest by pressure with her hands; worse at night, as it prevents all sleep, and feels in the morning unrefreshed and bruised all over; prostration and exhaustion. One solitary dose of Ammonium-brom. 3d cent., removed the whole trouble as if by magic, and the old lady is happy again. Eichler led the attention, years ago, to Ammonium-bromatum and Iodatum, where copious mucus is produced from the nostrils or from the respiratory organs, but so far very little clinical experience has been recorded, either from negligence or from failure in its application. Goullon gives us the following valuable hints for its use:

1st. Chronic bronchial catarrh, turning into an acute stage.

2d. Constant rattling in chest, and still difficult painful cough, partially and momentarily relieved by pressure of hands upon the chest.

3d. Insomnia from the constant cough and desire to expectorate.

4th. Hebetude and exhaustion from the constant cough.—*Pap. Zeitschrift*, 16, 91.

